# Can we enhance trust in the circular economy through referral marketing control?

**DOI:** 10.1371/journal.pone.0332348

**Published:** 2025-09-22

**Authors:** Deborah Lacitignola, Angela Martiradonna

**Affiliations:** 1 Dipartimento di Ingegneria Elettrica e dell’Informazione, Università di Cassino e del Lazio Meridionale, Cassino (FR), Italy; 2 Department of Economics, University of Foggia, Foggia, Italy; Universita degli Studi di Trieste, ITALY

## Abstract

Referral marketing can be considered a promising tool to support the transition toward a circular economy, thanks to its capacity to raise consumer awareness, foster loyalty and encourage participation in sustainable practices. However, to strengthen public trust in new circular business models, it becomes essential to manage such campaigns carefully. In this paper, we apply the Z-control method to regulate the dynamics of a referral marketing model with self-information, through control actions capable of influencing a campaign’s persistence and preventing its risk of collapse. Our focus is on two types of direct interventions: increasing the engagement of broadcasters and reducing consumer inertia. The analysis highlights how these marketing strategies must be applied over time in order for circular products, practices and behaviors to gain stable traction among the population. Reducing inertia, in particular, emerges as the more difficult challenge, suggesting that companies may need to adopt stronger marketing efforts while being mindful of how and when such efforts are applied. In this context, a non-monotonic application of marketing interventions over time appears especially important to avoid overwhelming consumers early on and to better address the psychological resistance often linked to inertia.

## 1 Introduction and research motivations

Resource extraction, land-use changes, uncontrolled waste and emissions are pushing socio-ecological systems to exceed their adaptation limits, hence entering new, less favorable regimes of functioning. On the wave of this now undeniable evidence, Circular Economy (CE) has emerged as an alternative way of organizing industrial systems and continues to attract attention as a primary solution to keep socio-ecological systems within limits favorable for humankind [1,2]. As defined by the European Union, CE is a model of production and consumption based on the reuse and regeneration of materials or products, especially as a means of continuing production in a sustainable or environmentally friendly way [3]. Due to its constitutive features, it therefore represents a clear departure from the traditional linear economic model, instead based on the *take-make-dispose* pattern [4].

The transition toward CE can bring about a wide variety of benefits in many different directions, such as reducing harmful emissions released into the environment, increasing the availability of raw materials, reducing waste materials, increasing competitiveness within the market, and so on [5]. However, despite the improvements that the CE paradigm could bring to today’s production and consumption methods, its practical implementations continue to be too limited. The debate about why this model is not the predominant one is still ongoing with the aim of detecting the different causes underlying this evidence [6,7]. Although the focus has mainly been on technological or economic barriers, the role of the consumer is gaining increasing importance [8]. The consumer has in fact been recognized as one of the key factors that can hinder or promote the implementation of the circular model, since companies can be driven by the demand of consumers for sustainable products and services [9–12]. In this paper, we want to engage in this debate, focusing our attention on the possible role of referral marketing campaigns [13] in promoting the transition to CE. Due to its peculiar characteristics and because of the pivotal role that consumers play for the campaign’s success, referral marketing can be in fact seen as a promising opportunity for companies involved in the CE transition to promote products, services or technologies with low environmental impact. For this reason in particular, it becomes essential to ‘manage’ the referral campaign through marketing strategies suitably dosed over time in order to ensure its survival. In this paper, we will show that the Z-control approach is able to provide interesting insights in this regard.

A referral campaign is a marketing program that encourages existing customers to promote and recommend their products or services among their social contacts, typically in exchange for incentives or rewards. Its goal is to leverage the trust and relationships between customers and their networks to attract new customers. The strength of referral marketing lies in the fact that it is based on personal networks, where friends’ and peers’ recommendations are highly trusted and can lead to higher conversion rates compared to other marketing techniques [14]. This peculiarity makes referral marketing a valuable tool for boosting confidence in new circular business models. In addition, it offers the opportunity to educate customers by raising their awareness and engaging more people in the movement toward a circular, resource-efficient economy.

In order to describe the dynamics underlying the spread of a referral campaign, in [15,16] a mathematical model was proposed with the total population divided into three mutually exclusive subpopulations: the unaware, the broadcaster and the inert individuals. The information mechanism involved was essentially passive, because the spread of the message was based only on referrals. However, in a referral marketing campaign, the nature of information for the potential consumer can also be active and related to self-information activities [17]. To take this into account, in [18] the model was enriched with the self-information mechanism and the impact of the interaction between passive and active information on the survival chances of the referral campaign was investigated. The emergence of hysteretic phenomenology was found depending on the suitable interplay between the parameters that regulate the reciprocal transition between the broadcasters and the inert class. Anyway, the question of how to effectively ‘control’ the spread of the referral campaign remains an interesting open issue that should be explored further.

To fill this gap, in this paper, we aim to consider direct controls on specific class of individuals involved in the campaign and use the Z-control approach to detect what trend over time such controls must display in order that the campaign - and the related CE paths or adoptions - could become stably rooted in the population. The Z-type control approach is an error-based method proposed to solve dynamic problems [19,20] that, in the past decade, has expanded its range of applications, moving from engineering to life sciences problems [21–25]. This is closely related to classical nonlinear tracking control techniques based on error dynamics. From a theoretical perspective, it shares foundational principles with Lyapunov-based error control - where a control law is designed to ensure the convergence of an error function - and Input–Output Feedback Linearization, which seeks to linearize nonlinear systems through state feedback and coordinate transformation. These approaches have a long-standing tradition and are widely applied in engineering domains such as electric motor control and industrial robotics [26–28]. While the formulation and notational framework of Z-control may differ, its core mechanism - using error dynamics to influence system behavior - aligns with these well-established methodologies. The strength of the approach relies in fact on its interesting property of stabilizing the system dynamics towards a desired target. This is due to the ability of the Z-control to change the dynamics of the uncontrolled system, pushing it toward a desired controlled equilibrium that becomes the only attractor for the Z-controlled model [29].

To the best of our knowledge, the application of the Z-control approach to referral marketing campaigns in the context of CE transition has not yet been explored in the literature. We believe this perspective might provide interesting insights on possible strategies to apply in order to manage the campaign, and hence CE paths or adoptions, over time. Rather than aiming at an empirical investigation, this work wants to develop a theoretical framework based on nonlinear dynamical systems, with the goal of deriving qualitative insights into how marketing actions can be structured and timed to promote long-term engagement and prevent campaign collapse or hysteresis.

As a further step in our theoretical exploration, we also examine how the model behaves under bounded stochastic fluctuations affecting key parameters, in order to reflect the inherent uncertainty of socio-economic environments. In particular, we consider random variability in the reactivation rate, modeled through a sine-Wiener process, to assess the structural robustness of the system and the stability of the Z-controlled dynamics. This additional layer of analysis can offer deeper insight into the resilience of marketing interventions over time and their capacity to sustain trust and participation in circular economy practices even under uncertain conditions.

We finally stress that this paper is built around a question: *Can we enhance trust in the circular economy through referral marketing control?* Rather than proposing a definitive answer, we adopt a theoretical and exploratory perspective. On this line, CE is not treated as a background theme but as the motivating context where behavioral resistance, such as consumer inertia, poses significant challenges to adoption. Our aim is to contribute a modeling-based framework that opens new ground for understanding how controlled referral dynamics may help overcome such barriers and promote long-term engagement in CE practices.

The paper is structured as follows. In Sect 2, we present the uncontrolled referral model with self-information and its dynamics. In Sect 3, the Z-type control method is applied in order to suitably regulate the dynamics of the broadcaster and inert classes. The case of direct Z-control on broadcasters and on inerts has been considered separately and the related Z-controlled models have been analytically investigated. Conditions expressed in terms of the system parameters have been obtained for boundedness and positiveness of solutions and the existence and stability properties of the Z-controlled equilibria have been proven. In Sect 4, through numerical investigations, we validate the above theoretical results and gain insight into the trend that the Z-controllers must display over time to effectively manage the referral marketing campaign. Moreover, in Sect 5, we explore the effect of bounded stochastic fluctuations on the system’s dynamics and control strategies. The results obtained are discussed in Sect 6, with some managerial implications and future perspectives outlined in Sect 7.

## 2 The uncontrolled model: A brief overview

In this section, we introduce the referral model with self-information that we will indicate as *the uncontrolled model* and present a brief overview of the main results obtained in [18]. Building on previous epidemiologically-inspired referral marketing models [15,16], the referral model with self-information proposed in [18] includes the role of consumers in actively searching and using product information, hence going beyond the mere word-of-mouth transmission. From a mathematical perspective, this is represented through a delay-based self-information mechanism inspired by epidemiological modeling literature [30] and adapted here to the marketing context to reflect how consumers’ past experiences influence their current decisions. Although active information seeking is not exclusive to marketing and also appears in other fields such as, for example, in vaccination models that incorporate information, where individuals assess risks and benefits before deciding to vaccinate [30–32], referral marketing is distinctively characterized by the combined interplay of: (i) social referral dynamics (word-of-mouth spread); (ii) consumer active learning and evaluation (self-information); (iii) the possibility of ‘reactivation’ of inert individuals through social influence. Together, these features allow the model to more realistically represent the complex interplay of trust, satisfaction and decision-making that drives successful referral campaigns. The model is governed by the following system of nonlinear differential equations:

$$\dot{u}=\mu -\rho \;b\;u-\mu \;u-\gamma \;m\;u,\;\dot{b}=p\;\rho \;b\;u-\sigma \;b+\alpha \;p\;b\;i-\mu \;b+\lambda \;i+\gamma \;q\;m\;u,\;\dot{i}=(1-p)\;\rho \;b\;u+\sigma \;b-\alpha \;p\;b\;i-\lambda \;i-\mu \;i+\gamma \;(1-q)\;m\;u,\;\dot{m}=a\;k\;b-a\;m,$$ (1)

where the state variables *u*, *b* and *i* respectively represent the fraction of the unaware, broadcaster and inert classes normalized by the total population. Unaware individuals are potential consumers who have not yet been exposed to the marketing message; broadcasters are individuals who have received the message and are actively spreading it to others, whereas inert individuals have encountered the message but choose not to spread it further. The self-information variable *m* summarizes information about the product performance, based on the past experiences of the customers in the recent past. For details on the self-information mechanism and the related modeling, refer to [18].

In the model, unaware individuals receive referral messages through interactions with broadcasters at a rate *ρ* and they may transition to the broadcaster class with probability *p* or become inert with probability 1–*p*. Broadcasters can stop disseminating the message and move to the inert class at a rate *σ*, or reactivate previously inert individuals through interactions at a reactivation rate *α*. Inert individuals, although unengaged, can become broadcasters due to external influences at a rate *λ*. Due to self-information, unaware individuals can exit the unaware class at a rate *γ*, moving to the broadcaster class with probability *q* and to the inert class with probability 1–*q*. The meaning of the involved parameters can therefore be summarized as follows: *ρ* represents the rate at which referral messages spread, *p* denotes the level of trust in the source, *σ* is the dropout rate of broadcasters in favor of the inert class, *α* is the reactivation rate from the inert to the broadcaster class, *λ* enables reactivation of inert individuals due to external factor, *γ* governs the effect of self-information, *k* determines the influence of past consumer behavior, and *a* dictates the decay of memory effects. The parameter *q* represents the level of customer satisfaction derived from self-information, influencing the likelihood that an unaware individual transitions to a broadcaster rather than becoming inert.

The total population density n(t)=u(t)+b(t)+i(t) follows the law


$$\dot{n}=\mu \;(1-n).$$

When μ=0, *n*(*t*) remains constant over time. In this scenario, the total population is not affected by external growth or decline and evolves only through internal redistribution among the different groups.

**Fig 1:**
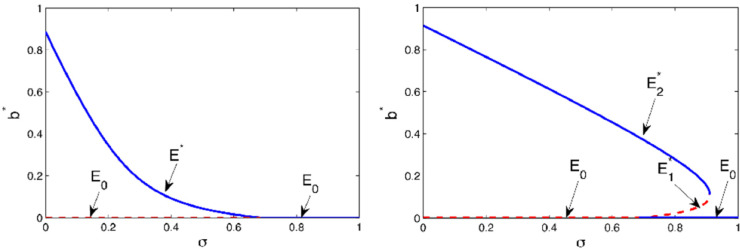
Bifurcation diagram in the plane ($\sigma ,b^{*}$). The other parameters are μ=0.05; ρ=0.25; λ=0.02; *p* = 0.7; *q* = 0.8; *a* = 0.5; *k* = 2; γ=0.2 so that $\alpha^{*}=1.1684$ and $\sigma_{c}=0.6850$. The solid lines (-) denote stability; the dashed lines (- -) denote instability. (Left) Forward scenario. The case $\alpha <\alpha^{*}$, α=0.4 - At $\sigma =\sigma_{c}=0.6850$, system (1) exhibits a forward bifurcation. (Right) Backward scenario. The case $\alpha >\alpha^{*}$, α=2 - At $\sigma =\sigma_{c}=0.6850$, system (1) exhibits a backward bifurcation. The value $\sigma_{SN}=0.9105$ is the saddle-node bifurcation threshold. Please refer to [18] for the analytical expressions of these bifurcation thresholds.

The uncontrolled system always admits a campaign-free equilibrium *E*_0_ = (1,0,0,0) which means that, without active broadcasters, the marketing message stops spreading. However, the model also supports campaign-standing equilibria $E^{*}=(u^{*},b^{*},i^{*},m^{*})$ where the referral campaign survives, since a fraction of the population is still actively involved in the spread of the message. The bifurcation analysis proposed in [18] highlights the emergence of two possible scenarios for message propagation. In fact, both forward and backward phenomenology was observed. As shown in Fig 1, their occurrence essentially depends on the suitable interplay between the two parameters *α* and *σ* that regulate the reciprocal transition between the broadcaster and the inert classes through referrals.

If marketing strategies lead to a reactivation rate *α* below a certain threshold $\alpha^{*}$, then a forward scenario is found, Fig 1 (left). In this case, values of the dropout rate *σ* that exceed the transcritical bifurcation value $\sigma_{c}$ have the effect of stopping the campaign and, to recover a campaign-standing situation, the value of *σ* must be simply reduced below the critical value $\sigma_{c}$. In contrast, when marketing efforts ensure sufficiently higher values of the reactivation rates ($\alpha >\alpha^{*}$), a backward scenario occurs. In this case, simply increasing the parameter *σ* above the threshold $\sigma_{c}$ is not enough to stop the campaign. To this aim, the values of *σ* must exceed the saddle-node bifurcation value $\sigma_{SN}>\sigma_{c}$.

Even if at first glance the occurrence of a backward scenario would seem to increase the campaign’s chances of survival, it however introduces a factor of risk. This is because, for $\sigma_{c}<\sigma <\sigma_{SN}$, the system exhibits a bistability situation with both the campaign-free equilibrium and the campaign-standing equilibrium as local attractors. In this bistability range, the dynamics of the system is highly dependent on the initial conditions and hysteresis-type behaviors can emerge, hence leading to a sudden stop of the campaign. In this case, reducing *σ* below the saddle-node bifurcation threshold $\sigma_{SN}$ is not enough to recover a campaign-standing situation and the parameter *σ* must be further reduced below the transcritical bifurcation threshold $\sigma_{c}$. This means that within the backward framework, once the message has stopped spreading, restoring it requires significantly greater marketing investment than what was initially needed to sustain the campaign. A backward scenario hence causes the spread of the referral message to follow a path less controllable and definitely more difficult to manage compared to the forward case. While the bifurcation structure of the referral model with self-information preserves its qualitative features - namely the presence of forward and backward bifurcation scenarios - even in the absence of self-information as in [15,16], the introduction of the self-information mechanism significantly affects the thresholds at which these bifurcations occur. More precisely, parameters related to self-information modulate the behavior of the system by mitigating undesired backward regimes and promoting the more desirable forward scenario. This peculiar role of self-information was thoroughly analyzed in [18], where existing referral marketing models [15,16] were extended to incorporate active consumer learning and evaluation processes beyond passive word-of-mouth transmission. The analysis revealed that intermediate levels of self-information can *optimize* campaign sustainability by enlarging the parameter regions associated with a single stable campaign-standing equilibrium and by stabilizing the forward regime. On the contrary, excessively low or high levels of self-information can lead to bistability, hence exposing the campaign to hysteretic dynamics and sudden collapses. In this sense, suitable levels of self-information can transform potential risks associated with referral campaigns into strategic opportunities for more predictable and stable viral marketing outcomes. This insight highlights the importance of integrating active information-seeking behaviors in referral marketing models to more accurately capture and influence real-world consumer dynamics. However, although self-information can play a crucial role in mitigating the risk of sudden collapses in engagement [18], a more effective strategy is needed to definitely eliminate the emergence of hysteretic phenomena in the campaign. In this respect, due to its ability to prevent backward bifurcation scenarios and to drive the system dynamics toward a desired target [22], the Z-control approach represents an interesting opportunity that we want to explore more thoroughly.

In the next section, we hence apply the Z-control method to suitably regulate the dynamics of the broadcaster and inert classes through control actions that can influence campaign persistence and prevent the risk of collapse. As just discussed above, the Z-control approach provides a structured approach to steering a system towards a desired state since, by forcing specific error terms to decay exponentially over time, it ensures that the controlled variables reach and maintain predefined values.

## 3 The Z-controlled model

Direct control of broadcasters or inerts can be implemented through targeted marketing interventions designed in such a way as to both increase broadcaster involvement and reduce inertia. For the sake of clarity, we point out that in this work, the term ‘inertia’ is used to indicate the size of the inert population namely the group of individuals that do not participate in the referral campaign because of psychological or social resistance. This kind of control can be based on suitable incentives, a good user experience and a consistent communication like frequent updates, personalized reminders, re-engagement campaigns. To increase the involvement of broadcasters while decreasing inertia, we consider the system of differential equations that govern the referral marketing dynamics (1) and introduce the time-dependent Z-control function *G*(*t*) whose dynamics is not prescribed a priori. This leads to the controlled system:

$$\dot{u}=\mu -\rho \;b\;u-\mu \;u-\gamma \;m\;u,\;\dot{b}=p\;\rho \;b\;u-\sigma \;b+\alpha \;p\;b\;i-\mu \;b+\lambda \;i+\gamma \;q\;m\;u+G(t)\;b,\;\dot{i}=(1-p)\;\rho \;b\;u+\sigma \;b-\alpha \;p\;b\;i-\lambda \;i-\mu \;i+\gamma \;(1-q)\;m\;u-G(t)\;b,\;\dot{m}=a\;k\;b-a\;m.$$ (2)

### 3.1 Direct Z-control on broadcasters

If the control function *G*(*t*) in (2) is introduced with the objective of setting the broadcaster level at a desired value *b*_*d*_, then it is designed so that the deviation from *b*_*d*_ decays exponentially over time. Applying the Z-control methodology, we define the tracking error:


$$e_{b}(t)=b(t)-b_{d},$$

which is enforced to follow the condition:


$${\dot{e}}_{b}+\xi e_{b}=0,\;\xi >0,$$

leading to the control law:

$$\dot{b}=-\xi (b-b_{d}).$$ (3)

The strictly positive parameter ξ - also known as the design parameter - indicates the convergence rate and gives a measure of the strength of the exerted control. By equating this with the controlled Eq (2), we obtain

$$G(t)=-p\;\rho \;u(t)-\alpha \;p\;i(t)+\sigma +\mu -\frac{\gamma \;q\;m(t)\;u(t)+\lambda \;i(t)+\xi \;(b(t)-b_{d})}{b(t)},$$ (4)

provided that *b*(*t*) > 0 for all t≥0. This control function ensures that the broadcaster population *b*(*t*) converges exponentially to the desired value *b*_*d*_.

By substituting the expression for *G*(*t*) into the equation governing *i*, we obtain:

$$\dot{i}=\rho \;b\;u-\mu \;b+\gamma \;m\;u-\mu \;i+\xi (b-b_{d}).$$ (5)

Thus, whenever *b*(*t*) > 0, the Z-controlled model (2) is equivalent to:

$$\dot{u}=\mu -\rho \;b\;u-\mu \;u-\gamma \;m\;u,\;\dot{b}=-\xi (b-b_{d}),\;\dot{i}=\rho \;b\;u+\gamma \;m\;u-\mu \;b-\mu \;i+\xi \;(b-b_{d}),\;\dot{m}=a\;k\;b-a\;m.$$ (6)

The total population density, defined as n(t)=u(t)+b(t)+i(t), follows the equation:

$$\dot{n}=\mu (1-n).$$ (7)

and the interval [0,1] is invariant under the dynamics of *n*(*t*). Moreover, in the special case where μ=0, the equation simplifies to $\dot{n}=0$, implying that the total population density remains constant over time.

Notice that the equivalence between the systems (2) and (6) is maintained under the assumption that the initial condition on *b* satisfies *b*_0_>0. If *b*_0_ = 0, the equation for *b* in (6) would reduce to $\dot{b}=\xi b_{d}$, even though the control function *G*(*t*) in (2) is not defined. Moreover, if *b*_0_>0, then the solution for *b*(*t*) is:

$$b(t)=b_{d}+(b_{0}-b_{d})e^{-\xi t},$$ (8)

which remains strictly positive for all t≥0. This ensures that *G*(*t*) is always well-defined and that the two systems remain equivalent. Furthermore, to improve the performance of the marketing campaign, we assume $b_{d}>b_{0}>0$, ensuring that the control mechanism actively drives the system towards a higher population of broadcasters over time.

#### 3.1.1 Boundedness and positivity of solutions.

In order to obtain the boundedness and positivity of solutions, we prove the following result:

**Proposition 1.** *Let the initial conditions u*(0) = *u*_0_, *b*(0) = *b*_0_, *i*(0) = *i*_0_, *m*(0) = *m*_0_, *satisfy:*


$$0\leq u_{0}\leq 1,\;0<b_{0}<b_{d},\;0\leq i_{0}\leq 1,\;u_{0}+b_{0}+i_{0}\leq 1,\;m_{0}\geq 0$$

*Then, the following bounds hold:*


$$u_{1}\leq u(t)\leq 1,\;b_{0}\leq b(t)<b_{d},\;i(t)\leq 1,\;u(t)+b(t)+i(t)\leq 1,\;m_{1}\leq m(t)<m_{2}$$

*for all t*, *where:*


$$m_{1}=\min(m_{0},k\;b_{0}),\;m_{2}=\max(m_{0},k\;b_{d}),\;u_{1}=\min\left(u_{0},\frac{\mu }{\mu +\rho \;b_{d}+\gamma \;m_{2}}\right).$$

*Moreover, if*

$$(\rho \;b_{0}+\gamma \;m_{1})\;u_{1}-\mu \;b_{0}>0,$$ (9)

*then, in correspondence of*

$$\xi \leq \xi_{max}=\frac{(\rho \;b_{0}+\gamma \;m_{1})\;u_{1}-\mu \;b_{0}}{b_{d}-b_{0}},$$ (10)

*we have that* i(t)≥0 *for all t*.

*Proof*: Since $0<b_{0}<b_{d}$, from (8), *b*(*t*) grows exponentially from *b*_0_ to *b*_*d*_, hence $b_{0}\leq b(t)<b_{d}$ for every *t*. Taking into account the differential equation for *m*: $\dot{m}=a\;k\;b\;-\;a\;m$, we obtain the bounds


$$a\;k\;b_{0}-a\;m\leq \dot{m}<a\;k\;b_{d}-a\;m.$$

Using a comparison argument, it follows that


$$m_{1}=\min(m_{0},k\;b_{0})\leq m(t)\leq \max(m_{0},k\;b_{d})=m_{2}.$$

For the differential equation governing *u*, we have the following bound for $\dot{u}$:


$$\dot{u}\geq \mu -(\mu +\rho \;b_{d}+\mu +\gamma \;m_{2})\;u.$$

By applying the comparison principle, we conclude that $u(t)\geq u_{1}.$ When *u* = 1, we have $\dot{u}=-\rho \;b\;-\;\gamma \;m<0,$ which implies that the solution crosses the manifold *u* = 1 with a negative derivative. Hence, by the maximum principle, *u*(*t*) cannot exceed 1.

Moreover, the function n(t)=u(t)+b(t)+i(t) verifies the Eq (7), which ensures that 0≤n(t)≤1. Since b(t),u(t)≥0, it follows directly that i(t)≤n(t)≤1. Finally, we evaluate the equation for *i*. When *i* = 0, using the bounds on *b*, *m*, and *u*, along with condition (10), we obtain:


$$\dot{i}\geq \rho \;b_{0}\;u_{1}+\gamma \;m_{1}\;u_{1}-\mu \;b_{d}+\xi \;(b_{0}-b_{d})\geq 0.$$

This implies that *i*(*t*) crosses the manifold *i* = 0 with a non-negative derivative, ensuring that *i*(*t*) remains non-negative for all *t*. □

We note that Proposition 1 provides a range in which the parameter ξ must vary (i.e. $\xi \in (0,\xi_{max}]$ in order to ensure the positiveness of *i*(*t*) for all *t* > 0. Therefore, for a successful application of the Z-control approach, the strength of the control needs to be carefully calibrated. It is important to note that the feasibility of such a range depends on the positivity of the threshold $\xi_{\max}$, which is itself guaranteed by the validity of condition (9). To facilitate verification of this condition, we provide the following remark, which identifies explicit parameter regimes under which $\xi_{max}>0$.

**Remark 1.** *Assume that* μ<ρ+γk *and* $b_{d}<1-\frac{\mu }{\rho +\gamma \;k}$. *Condition* (9) *holds in one of the following cases:*

*(i)* $\frac{b_{0}}{\gamma }\;\left(\mu -\rho +(\rho +\gamma \;k)\;b_{d}\right)\leq m_{0}\leq k\;b_{0}\;\wedge \;u_{0}>\frac{\mu \;b_{0}}{\rho \;b_{0}+\gamma \;m_{0}}$;
*(ii)* $k\;b_{0}<m_{0}<\frac{1}{\gamma }\left(\rho \;(1-b_{d})+\gamma \;k-\mu \right)\;\wedge \;u_{0}>\frac{\mu }{\rho +\gamma \;k}$.

*Proof*: We analyze the two cases separately. In both cases, we refer to the quantities *m*_1_, *m*_2_, and *u*_1_ as defined in Proposition 1.

In case , from the assumed conditions, we obtain:


$$m_{1}=m_{0},\;m_{2}=k\;b_{d},\;u_{1}=\min\left(u_{0},\frac{\mu }{\mu +(\rho +\gamma \;k)\;b_{d}}\right).$$

We now distinguish two subcases. If $u_{0}\leq \frac{\mu }{\mu +(\rho +\gamma \;k)\;b_{d}}$, then $u_{1}=u_{0}$, and condition (9) becomes:


$$(\rho \;b_{0}+\gamma \;m_{1})\;u_{1}-\mu \;b_{0}=(\rho \;b_{0}+\gamma \;m_{0})\;u_{0}-\mu \;b_{0}>0,$$

which holds by assumption. Otherwise, we have $u_{1}=\frac{\mu }{\mu +(\rho +\gamma \;k)\;b_{d}}$ and condition (9) becomes:


$$(\rho \;b_{0}+\gamma \;m_{1})\;u_{1}-\mu \;b_{0}=\mu \;\frac{\gamma \;m_{0}-b_{0}\;(\mu -\rho +(\rho +\gamma \;k)\;b_{d})}{\mu +(\rho +\gamma \;k)\;b_{d}}>0,$$

which again holds by assumption.

In case , from the assumed conditions we obtain $m_{1}=k\;b_{0}$. We also note that, under the hypotheses μ<ρ+γk and $b_{d}<1-\frac{\mu }{\rho +\gamma \;k}$, the upper bound for *m*_0_ is positive. Again,we now distinguish two subcases. If $u_{0}\leq \frac{\mu }{\mu +\rho \;b_{d}+\gamma \;m_{2}}$, then $u_{1}=u_{0}$ and


$$(\rho \;b_{0}+\gamma \;m_{1})\;u_{1}-\mu \;b_{0}=b_{0}((\rho +\gamma \;k)\;u_{0}-\mu )>0;$$

then condition (9) is verified. Otherwise, $u_{1}=\frac{\mu }{\mu \;+\;\rho \;b_{d}+\gamma \;m_{2}}$ and


$$(\rho \;b_{0}+\gamma \;m_{1})\;u_{1}-\mu \;b_{0}=\left\{ \begin{array}{cc} \mu \;b_{0}\;\frac{\rho (1-b_{d})+\gamma \;k-\mu -\gamma \;m_{0}}{\mu +\rho \;b_{d}+\gamma \;m_{0}}>0, & \text{if }m_{0}\geq k\;b_{d}\\ \mu \;b_{0}\;\frac{(\rho +\gamma \;k)\;(1-b_{d})-\mu }{\mu +\rho \;b_{d}+\gamma \;k\;b_{d}}>0, & \text{if }m_{0}<k\;b_{d} \end{array}\right.$$

In both cases, the positivity of the expression is ensured, thereby verifying condition (9). □

In summary, Proposition 1 and the subsequent remark offer sufficient conditions under which the positivity and boundedness of the model solutions are preserved. This ensures not only the mathematical well-posedness of the controlled dynamics but also the practical feasibility of the proposed control in a marketing context.

#### 3.1.2 The Z-controlled equilibrium and its local stability properties.

We are now ready to show that the Z-controlled model admits a unique Z-controlled equilibrium that is locally asymptotically stable.

**Proposition 2.** *Assume that* μ<ρ+γk, $b_{0}<b_{d}<1\;-\;\frac{\mu }{\rho +\gamma \;k}$ *and* ξ>0. *Then the Z-controlled system* (6) *admits only one feasible equilibrium* $E^{*}=(u^{*},b^{*},i^{*},m^{*}),$ *where*


$$u^{*}=\frac{\mu }{\mu +(\rho +\gamma k)b_{d}},\;b^{*}=b_{d},\;i^{*}=b_{d}\frac{(\rho +\gamma k)(1-b_{d})-\mu }{\mu +(\rho +\gamma k)b_{d}},\;m^{*}=kb_{d}.$$

Moreover, E^*^ *is locally asymptotically stable.*

*Proof*: It is immediate to verify that (u^*^,b^*^,i^*^,m^*^) is the unique solution of the steady-state system


$$\mu -\rho \;b\;u-\mu \;u-\gamma \;m\;u=0,\;\xi (b-b_{d})=0,\;\rho \;b\;u+\gamma \;m\;u-\mu \;b-\mu \;i+\xi \;(b-b_{d})=0,\;a\;k\;b-a\;m=0.$$

Therefore, E^*^ is the unique equilibrium point of the Z-controlled system (6). Under the stated assumptions, one easily checks that $u^{*},b^{*},i^{*}\in [0,1]$ and $m^{*}\geq 0$, ensuring that the equilibrium is biologically feasible. As far as the stability analysis is concerned, the Jacobian matrix of the system evaluated at E^*^ is given by


$$J^{*}=(\begin{array}{cccc} -\mu -(\rho +\gamma \;k)\;b_{d} & -\rho \;u^{*} & 0 & -\gamma \;u^{*}\\ 0 & -\xi & 0 & 0\\ (\rho +\gamma \;k)\;b_{d} & \xi -\mu & -\mu & \gamma \;u^{*}\\ 0 & a\;k & 0 & -a \end{array})$$

whose eigenvalues are:


$$-\mu -(\rho +\gamma k)\;b_{d},\;-\xi ,\;-\mu ,\;-a.$$

Since all eigenvalues are negative when ξ>0, the equilibrium point E^*^ is locally asymptotically stable. □

This result confirms that the Z-control mechanism effectively regulates the broadcaster population *b*(*t*), driving it exponentially toward the desired target level *b*_*d*_. The existence and local stability of the corresponding controlled equilibrium ensure that the system maintains a consistent and stable broadcasting intensity over time, which is a key aspect for the success and sustainability of the marketing campaign.

### 3.2 Direct Z-control on inerts

We now consider an alternative Z-control strategy that aims to reduce the proportion of individuals who remain inert throughout the marketing process. If the control function *G*(*t*) in (2) is introduced with the objective of setting the inerts to a desired value *i*_*d*_, then *G*(*t*) is designed so that the deviation from *i*_*d*_ decays exponentially over time. Applying the Z-control methodology, we define the tracking error:


$$e_{i}(t)=i(t)-i_{d},$$

which is imposed to follow the dynamic equation:


$${\dot{e}}_{i}+\zeta e_{i}=0,\;\zeta >0.$$

This leads to the control law:

$$\dot{i}=-\zeta (i-i_{d}),$$ (11)

where the strictly positive parameter ζ represents the rate of convergence and quantifies the intensity of the applied control. By incorporating this equation into the controlled system (2), we derive:

$$G(t)=(1-p)\;\rho \;u(t)+\sigma -\alpha \;p\;i(t)+\frac{\gamma \;(1-q)\;m(t)\;u(t)-(\lambda +\mu )\;i(t)+\zeta (i(t)-i_{d})}{b(t)}$$ (12)

provided that *b*(*t*) > 0 for all t≥0. This control mechanism ensures that the inert population *i*(*t*) follows an exponential trajectory towards the target value *i*_*d*_.

To improve the effectiveness of the marketing campaign, we assume


$$0<i_{d}<i_{0},$$

which ensures that controlled dynamics lead the inert population *i*(*t*) to a lower equilibrium level. This assumption implies a reduction in the number of inactive individuals, leading to increased engagement and enhanced message diffusion.

Substituting the expression for G(t) into the governing equation for *b*, we derive:

$$\dot{b}=\rho \;b\;u+\gamma \;m\;u-\mu \;b-\mu \;i+\zeta (i-i_{d}),$$ (13)

Thus, considering the function defined in (12), whenever *b*(*t*) > 0, the Z-controlled model (2) is reformulated as:

$$\dot{u}=\mu -\rho \;b\;u-\mu \;u-\gamma \;m\;u\;\;\dot{b}=\rho \;b\;u+\gamma \;m\;u-\mu \;b-\mu \;i+\zeta (i-i_{d}),\;\dot{i}=-\zeta (i-i_{d}),\;\dot{m}=a\;k\;b-a\;m.$$ (14)

As in Sect 3.1, the total population density, defined as n(t)=u(t)+b(t)+i(t), follows Eq (7), and the interval [0,1] remains invariant under its dynamics. Moreover, in the special case where μ=0, the total population density remains constant over time.

The equivalence between systems (2) and (14), with *G*(*t*) defined as in (12), holds if the initial condition on *b* satisfies *b*_0_>0 and *b*(*t*) remains positive for all *t*. In the following, we derive the conditions that guarantee this property.

#### 3.2.1 Boundedness and positivity of solutions.

To ensure that the Z-control strategy applied to the inert population is mathematically well-posed and biologically meaningful, we derive sufficient conditions under which the solutions of the controlled system remain positive and bounded for all t≥0. In particular, we show that all state variables stay within biologically admissible ranges, meaning that their values remain non-negative and do not exceed the maximal density threshold of 1.

**Proposition 3.** *Let the initial conditions u*(0) = *u*_0_, *b*(0) = *b*_0_, *i*(0) = *i*_0_, *m*(0) = *m*_0_, *satisfy:*


$$0\leq u_{0}\leq 1,\;0<b_{0}\leq 1,\;0\leq i_{d}<i_{0}\leq 1,\;u_{0}+b_{0}+i_{0}=1,\;m_{0}\geq 0,$$

and assume that

$$\rho \;b_{0}\;u_{0}-\mu \;(b_{0}+i_{0})>0.$$ (15)

*Then, for any* ζ>0, *the following bounds hold for all* t≥0:


$$0\leq u(t)\leq u_{0},\;b_{0}<b(t)\leq 1,\;i_{d}<i(t)\leq i_{0},\;u(t)+b(t)+i_{0}=1,\;m(t)\geq 0.$$

*Proof*: We start by observing that the solution of $\dot{i}=-\zeta (i-i_{d})$ is $i(t)=i_{d}\;+\;(i_{0}-i_{d})e^{-\zeta t}$, which ensures $i_{d}<i(t)\leq i_{0}$ for all t≥0. Concerning *u*(*t*), we note that at *u* = 0, we have $\dot{u}=\mu >0$, ensuring that *u*(*t*) cannot become negative. Moreover, the function n(t)=u(t)+b(t)+i(t) verifies $\dot{n}=0$, ensuring that u(t)+b(t)+i(t)=1 for all t≥0. Using this relation, the equation in *b* reduces to:


$$\dot{b}=\rho \;b\;(1-b-i)+\gamma \;m\;(1-b-i)-\mu \;b-\mu \;i+\zeta (i-i_{d}).$$

At this point, our aim is to prove that *b*(*t*)>*b*_0_ and *m*(*t*) > 0 for all t≥0. Suppose there exists a time *t*_1_>0 such that either $b(t_{1})=b_{0}$ or *m*(*t*_1_) = 0. We distinguish two cases

(i) $b(t_{1})=b_{0}$ and $m(t_{1})\geq 0$,
(ii) $b(t_{1})\geq b_{0}$ and *m*(*t*_1_) = 0,

that we analyze separately. In case (i), evaluating the equation for $\dot{b}$ at *t*_1_ and using the upper bound $i(t_{1})\leq i_{0}$, along with condition (15), we estimate:


$$\dot{b}(t_{1})\geq \rho \;b_{0}\;(1-b_{0}-i_{0})+\gamma \;m(t_{1})\;(1-b_{0}-i_{0})-\mu \;(b_{0}+i_{0})\geq \rho \;b_{0}\;u_{0}-\mu \;(b_{0}+i_{0})>0$$

Since $\dot{b}(t_{1})>0$, it follows that *b*(*t*) is increasing at *t*_1_. Therefore, in this case, we can conclude that *b*(*t*)>*b*_0_ for all t≥0. In case (ii), evaluating the equation for $\dot{m}$ at *t*_1_, we obtain:


$$\dot{m}(t_{1})=a\;k\;b(t_{1})\geq a\;k\;b_{0}>0.$$

Since $\dot{m}(t_{1})>0$, it follows that *m*(*t*) is increasing at *t*_1_. Therefore, we conclude that *m*(*t*) > 0 for all t≥0. Finally, from u(t)+b(t)+i(t)=1, it follows that b(t)=1−u(t)−i(t)≤1 and $u(t)=1\;-\;b(t)\;-\;i(t)\leq 1\;-\;b_{0}\;-\;i_{0}=u_{0}.$ This confirms the bounds on *b*(*t*) and *u*(*t*), completing the proof. □

#### 3.2.2 The Z-controlled equilibria and their local stability properties.

We now investigate the existence and local stability of equilibrium points resulting from the application of the Z-control strategy targeting the inert population. In particular, we show that the system can admit multiple equilibria and characterize their stability properties.

In the following, we assume that μ<ρ+γk and chose $i_{0}<i_{c}$, where

$$i_{c}=\frac{\rho +\gamma \;k-\mu }{\rho +\gamma \;k}.$$ (16)

In addition, let the target level *i*_*d*_ for the inerts be such that $i_{d}<i_{0}$. Then the following result holds:

**Proposition 4.** *If the target level i*_*d*_ *is such that* $i_{d}<i_{d1}$, *with*

$$i_{d1}=\rho +\gamma \;k+\mu -2\sqrt{\mu \left(\rho +\gamma \;k\right)},$$ (17)

*then the Z-controlled system* (14) *admits two distinct positive Z-controlled equilibria E*_1_ *and E*_2_ *with opposite stability properties.*

*Proof*: The Z-controlled system (14) admits as Z-controlled equilibria $E^{*}=(u^{*},b^{*},i^{*},m^{*}),$ where


$$u^{*}=1-b^{*}-i_{d},\;i^{*}=i_{d},\;m^{*}=k\;b^{*}$$

and b^*^ is a positive solution of the following algebraic equation

$$P_{2}\;b^{2}+P_{1}\;b+P_{0}=0,$$ (18)

whose coefficients are given by:


$$P_{2}=\rho +\gamma \;k>0,$$

$$P_{1}=\mu -(\rho +\gamma \;k)\;(1-i_{d})=(\rho +\gamma \;k)\;(i_{d}-i_{c})<0$$

$$P_{0}=\mu \;i_{d}>0.$$

To determine their feasibility, we thus inspect the discriminant of the algebraic Eq (18), namely


$$\Delta_{P}=P_{1}^{2}-4\;P_{2}\;P_{0}=(\rho +\gamma \;k{)}^{2}(i_{d}-i_{c}{)}^{2}-4\;\mu \;i_{d}(\rho +\gamma \;k)$$

Specifically, $\Delta_{P}=(\rho +\gamma \;k)\left(Q_{2}\;i_{d}^{2}-2\;Q_{1}\;i_{d}+Q_{0}\right)$, where:


$$Q_{2}=\rho +\gamma \;k>0,$$

$$Q_{1}=(\rho +\gamma \;k)\;i_{c}+2\mu =\rho +\gamma \;k+\mu >0,$$

$$Q_{0}=(\rho +\gamma \;k)i_{c}^{2}=\frac{(\rho +\gamma \;k-\mu {)}^{2}}{\rho +\gamma \;k}>0.$$

The discriminant of the quadratic expression within $\Delta_{P}$ is:


$$\Delta_{Q}=4(Q_{1}^{2}-Q_{2}\;Q_{0})=16\;\mu \left(\rho +\gamma \;k\right)>0,$$

which indicates that the equation $\Delta_{P}=0$ has two positive real roots $0<i_{d1}<i_{d2}$, given by:


$$i_{d1}=\rho +\gamma \;k+\mu -2\sqrt{\mu \left(\rho +\gamma \;k\right)},\;i_{d2}=\rho +\gamma \;k+\mu +2\sqrt{\mu \left(\rho +\gamma \;k\right)}.$$

Since $i_{d}<i_{d1}$, the discriminant $\Delta_{P}$ is positive, and Eq (18) has two distinct positive real roots $0<b_{1}<b_{2}<1$, given by:


$$b_{1/2}=\frac{-P_{1}\pm \sqrt{\Delta_{P}}}{2P_{2}}.$$

Therefore, two Z-controlled equilibria exist:


$$E_{1}=(1-i_{d}-b_{1},b_{1},i_{d},k\;b_{1}),\;E_{2}=(1-i_{d}-b_{2},b_{2},i_{d},k\;b_{2}),$$

where $1-i_{d}-b_{2}<1-i_{d}-b_{1}<1$. Ensuring feasibility of such equilibria requires $1-i_{d}-b_{2}>0$. To demonstrate this condition, we exploit the relation $P_{1}=\mu -P_{2}\;(i-i_{d})$, and observe that


$${\left((1-i_{d})\;P_{2}+\mu \right)}^{2}=\Delta_{P}+4\;P_{2}>\Delta_{P}.$$

Consequently, it follows that $(1-i_{d})\;P_{2}+\mu >\sqrt{\Delta_{P}}$. Now, expressing *b*_2_ as

$$b_{2}=\frac{(1-i_{d})P_{2}-\mu +\sqrt{\Delta_{P}}}{2P_{2}},$$ (19)

we can establish that:


$$1-i_{d}-b_{2}=\frac{1-i_{d}+\mu -\sqrt{\Delta_{p}}}{2P_{2}}>0.$$

Thus, the feasibility of the two equilibria $E_{1},\;E_{2}$ is ensured.

To analyze the local stability of *E*_1_ and *E*_2_, we consider the Jacobian matrix of the system evaluated at the equilibrium $E^{*}=(1-i_{d}-b^{*},b^{*},i_{d},k\;b^{*})$, where b^*^ is either *b*_1_ or *b*_2_:


$$J^{*}=(\begin{array}{ccccccc} -\mu -(\rho +\gamma \;k)\;b^{*} & & -\rho \;(1-i_{d}-b^{*}) & & 0 & & -\gamma \;(1-i_{d}-b^{*})\\ (\rho +\gamma \;k)\;b^{*} & & \rho \;(1-i_{d}-b^{*})-\mu & & -\mu +\zeta & & \gamma \;(1-i_{d}-b^{*})\\ 0 & & 0 & & -\zeta & & 0\\ 0 & & a\;k & & 0 & & -a \end{array})$$

The characteristic polynomial of J^*^ is given by:


$$L(\ell )=(\zeta +\ell )(\ell^{3}+L_{2}\ell^{2}+L_{1}\ell +L_{0}),$$

where the coefficients are defined as:


$$L_{2}=2\;\mu +a+(2\rho +\gamma \;k)\;b^{*}-\rho (1-i_{d}),$$

$$L_{1}=\mu^{2}+2a\;\mu -(\mu \;\rho +a\;(\rho +\gamma \;k))\;(1-i_{d})+(\mu \;(2\rho +\gamma \;k)+2a\;(\rho +\gamma \;k))\;b^{*},$$

$$L_{0}=a\;\mu \;\left(\mu -(\rho +\gamma \;k)\;(1-i_{d})+2(\rho +\gamma \;k)\;b^{*}\right).$$

Thus, the eigenvalues of J^*^ are −ζ and the solutions of the cubic equation:

$$\ell^{3}+L_{2}\ell^{2}+L_{1}\ell +L_{0}=0.$$ (20)

To determine stability properties, we apply the Routh-Hurwitz criterion on Eq (20). According to this criterion, stability is guaranteed if and only if the following conditions hold:


$$L_{2},\;L_{1},\;L_{0}>0\;\text{and}\;L_{1}L_{2}-L_{0}>0$$

If any of these conditions are violated, at least one root will have a positive real part, indicating linear instability. Exploiting the relation $P_{1}=\mu \;-\;P_{2}\;(i\;-\;i_{d})$, we can express the coefficients *L*_*i*_ in terms of *P*_1_ and *P*_2_ as:


$$L_{2}=a+2\;\mu +P_{2}\;b^{*}-\rho (1-i_{d}-b^{*}),$$

$$L_{1}=\mu^{2}+a\;\mu +a\;P_{1}+(\mu +2a)\;P_{2}\;b^{*}-\mu \;\rho \;(1-i_{d}-b^{*}),$$

$$L_{0}=a\;\mu \;\left(P_{1}+2P_{2}\;b^{*}\right).$$

Observe that, when $b^{*}=b_{1}=\frac{-P_{1}-\sqrt{\Delta_{P}}}{2P_{2}}$, the coefficient *L*_0_ becomes $L_{0}=-a\;\mu \;\sqrt{\Delta_{P}}<0,$ which implies the linear instability of the equilibrium *E*_1_. To assess the stability of equilibrium *E*_2_, we substitute $b^{*}=b_{2}$ from Eq (19) into the expressions for *L*_2_, *L*_1_, and *L*_0_, obtaining:


$$L_{2}=a+\frac{1}{2}\gamma \;k\;(1-i_{d})+\frac{\mu \;(2\rho +3\gamma \;k)+(2\rho +\gamma \;k)\;\sqrt{\Delta_{P}}}{2\;(\rho +\gamma \;k)}>0,$$

$$L_{1}=a\;\mu +2\;a\sqrt{\Delta_{P}}+\frac{1}{2}\mu \;\gamma \;k\;(1-i_{d})+\frac{\mu^{2}\;\gamma \;k+\mu \;(2\rho +\gamma \;k)\;\sqrt{\Delta_{P}}}{2(\rho +\gamma \;k)}>0,$$

$$L_{0}=a\;\mu \;\left(P_{1}+2P_{2}\;b_{2}\right)=a\;\mu \;\sqrt{\Delta_{P}}>0.$$

According to the the Routh-Hurwitz criterion, we now have to verify that $L_{1}L_{2}-L_{0}>0$. The following estimates hold for *L*_1_ and *L*_2_:

$$L_{2}>a+\frac{2\rho +\gamma \;k}{2(\rho +\gamma \;k)}\sqrt{\Delta_{P}},\;L_{1}>a\;\mu +\frac{\mu \;(2\rho +\gamma \;k)}{2(\rho +\gamma \;k)}\sqrt{\Delta_{P}},$$ (21)

from which we have that

$$L_{1}L_{2}>a\;\mu \;\frac{2\rho +\gamma \;k}{\rho +\gamma \;k}\sqrt{\Delta_{P}}>a\;\mu \;\sqrt{\Delta_{P}}=L_{0}.$$ (22)

Thus, all the Routh-Hurwitz conditions are verified and we can conclude that *E*_2_ is locally asymptotically stable. □

## 4 Numerical investigations

In this Section, we make use of the theoretical results obtained to gain insight into the trend that the Z-controller *G*(*t*) must show over time to ensure a successful Z-control of the referral marketing campaign. For the numerical investigations, we use the same parameters detailed in Fig 1 (μ=0.05, ρ=0.25, λ=0.02, *p* = 0.7, *q* = 0.8, *a* = 0.5, *k* = 2, γ=0.2) and estimated in [15,16] basing on data collected through an extensive questionnaire-based-survey. We choose as initial conditions *u*_0_ = 0.4; *b*_0_ = 0.5; *i*_0_ = 0.1; *m*_0_ = 1 to resemble a campaign that is initially characterized by a rather active involvement of broadcasters, a low level of inertia and a sufficiently high level of self-information.

We specifically want to determine whether the presence of a forward or a backward scenario in the uncontrolled model results in differences, in terms of the exerted control, when the long-run equilibrium of the uncontrolled model is *converted* into a new Z-controlled campaign-standing equilibrium.

**Fig 2:**
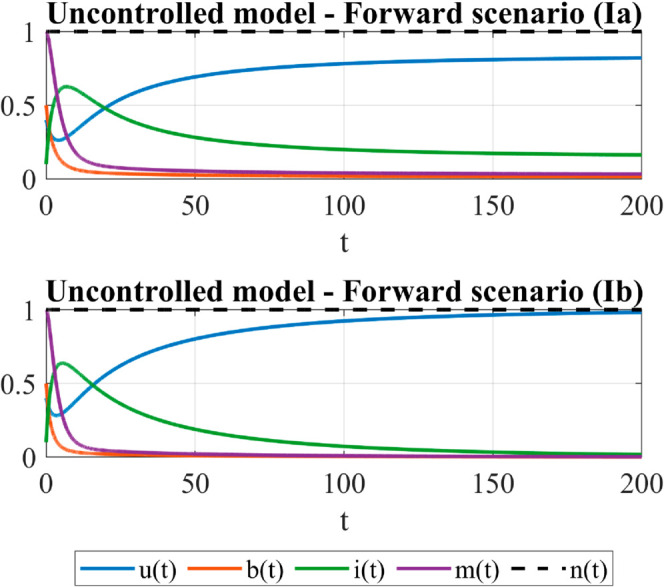
The forward scenario - Time dependent behavior of the uncontrolled model. Parameters: μ=0.05, ρ=0.25, λ=0.02, *p* = 0.7, *q* = 0.8, *a* = 0.5, *k* = 2, γ=0.2. For these values: $\alpha^{*}\approx 1.1684$, $\sigma_{c}\approx 0.6850$. Initial conditions: *u*_0_ = 0.4, *b*_0_ = 0.5, *i*_0_ = 0.1, *m*_0_ = 1. Case Ia: The forward scenario (subthreshold): $\alpha =0.4<\alpha^{*}$ and $\sigma =0.6<\sigma_{c}$. System trajectories approach the stable campaign-standing equilibrium $E^{*}\approx (0.8303,0.0157,0.1540,0.0314)$. Case Ib: The forward scenario (superthreshold): $\alpha =0.4<\alpha^{*}$, and $\sigma =0.8>\sigma_{c}$. System trajectories approach the campaign-free equilibrium *E*_0_ = (1,0,0,0).

To test the role of the control in the forward scenario, we consider both the case subthreshold (Ia: α=0.4 and σ=0.6 ) and superthreshold (Ib: α=0.4 and σ=0.8). In the former case, the uncontrolled model exhibits a stable campaign-standing equilibrium E^*^ substhreshold as the only system attractor, while in the latter one system trajectories approach the campaign-free equilibrium *E*_0_, Fig 1 (left). As Fig 2 (top) confirms, in the subcritical case (Ia) the dynamics of the uncontrolled model stabilizes on the campaign-standing equilibrium E^*^ which is, however, not particularly favorable since it is characterized by far fewer broadcasters, far more inerts and a very low level of self-information compared to the initial situation. In the supercritical case (Ib), as expected, the referral campaign stops spreading, Fig 2 (bottom).

**Fig 3:**
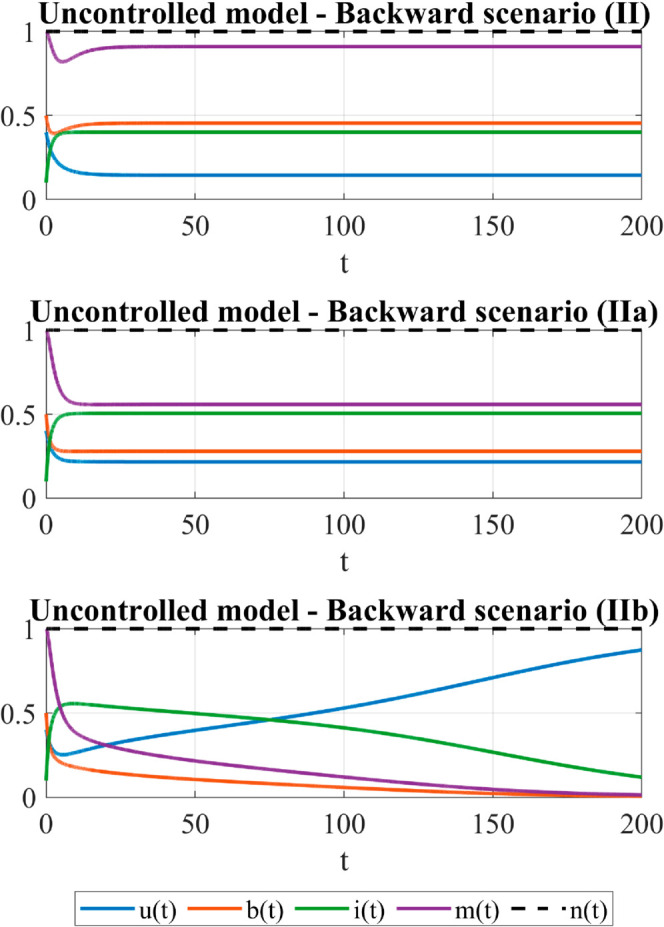
The backward scenario - Time dependent behavior of the uncontrolled model. Parameters: μ=0.05, ρ=0.25, λ=0.02, *p* = 0.7, *q* = 0.8, *a* = 0.5, *k* = 2, γ=0.2. For these values: $\alpha^{*}\approx 1.1684$, $\sigma_{c}\approx 0.6850$. Initial conditions: *u*_0_ = 0.4, *b*_0_ = 0.5, *i*_0_ = 0.1, *m*_0_ = 1. Case II: The backward scenario (subthreshold): $\alpha =2>\alpha^{*}$ and $\sigma =0.6<\sigma_{c}$. System trajectories approach the stable campaign-standing equilibrium $E^{*}\approx (0.1447,\;0.4548,\;0.4006,\;0.9096)$. Case IIa: The backward scenario (bistability): $\alpha =2>\alpha^{*}$ and $\sigma =0.8\in (\sigma_{c},\sigma_{SN})$. System trajectories approach the stable campaign-standing equilibrium $E^{*}=(0.2161,\;0.2790,\;0.5049,\;0.5580)$. Case IIb: The backward scenario (superthreshold): $\alpha =2>\alpha^{*}$ and $\sigma =0.95>\sigma_{SN}$. System trajectories approach the campaign-free equilibrium *E*_0_ = (1,0,0,0).

To investigate the role of the control on the backward scenario, we consider the subcritical case (II: α=2 and σ=0.6), the bistability case (IIa: α=2, σ=0.8) and the superthreshold case (IIb: α=2, σ=0.95). In the subcritical case, system trajectories of the uncontrolled model tends toward a campaign-standing equilibrium that is the only local attractor for the system; in case IIa, a bistability situation occurs so that both the campaign-standing equilibrium and the campaign-free equilibrium are local attractors of the system and system dynamics is highly dependent on initial conditions. In superthreshold case (IIb), the campaign-free equilibrium is the the only system attractor for the uncontrolled model, Fig 1 (right). In these cases and for initial conditions as above, the temporal evolution of the uncontrolled model is shown in Fig 3. We observe that in both the subcritical and the bistability case, the long-term equilibrium achieved in the uncontrolled case represents a significantly worse development of the campaign with respect to the initial situation, with a decrease in time of the activity level (fewer broadcasters) and an increase of the inertia (considerably more inerts). In the supercritical case (IIb), as expected, the referral campaign stops spreading.

### 4.1 Direct Z-control on the broadcasters

To ensure a better long-term situation compared to the initial one, the uncontrolled model can be Z-controlled with the aim of increasing the broadcasters to a desired value $b_{d}>b_{0}$. To do that, a suitable controller *G*(*t*) must be applied to the system with a strength ξ in $\left(0,\xi_{max}\right]$. For the chosen initial conditions and because of (10), in this case $\xi_{max}=0.0359$. We chose ξ=0.03 to ensure a sufficiently strong control on the campaign.

**Fig 4:**
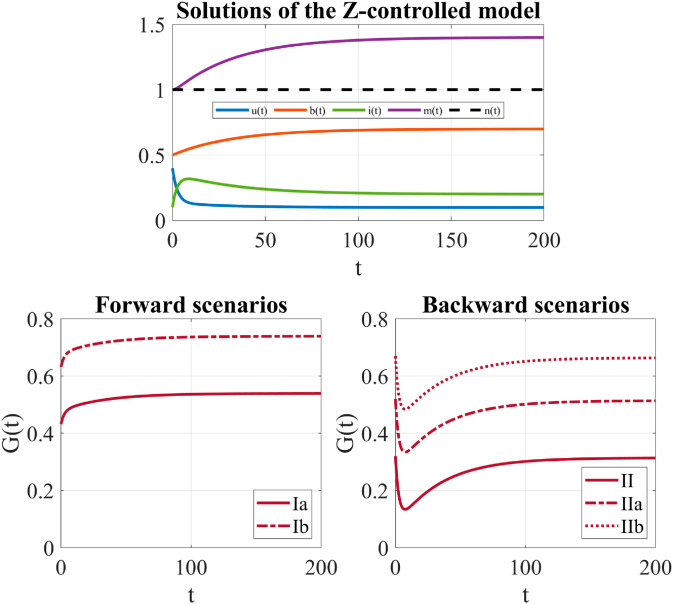
Z-controlled model: direct control on the broadcasters. Parameters: μ=0.05, ρ=0.25, λ=0.02, *p* = 0.7, *q* = 0.8, *a* = 0.5, *k* = 2, γ=0.2, ξ=0.03, and *b*_*d*_ = 0.7. Initial conditions: *u*_0_ = 0.4, *b*_0_ = 0.5, *i*_0_ = 0.1, $m_{0}=k\;b_{0}=1$. For these values, $\xi_{\max}\approx 0.0359$. (Top) System trajectories approach the Z-controlled equilibrium $E^{*}\approx (0.0990,\;0.7000,\;0.2010,\;1.4000)$. (Bottom) Control functions *G*(*t*) applied to convert an uncontrolled attractor within the forward or the backward scenario into the new Z-controlled equilibrium. Left: Forward scenario - The subthreshold case (Ia) and the superthreshold case (Ib). Right: Backward scenario - The subthreshold case (II), the bistability case (IIa) and the superthreshold case (IIb).

As shown in Fig 4 (Top), for both forward and backward scenarios, the Z-control forces the dynamics of the system to approach the Z-controlled campaign-standing equilibrium $E^{*}\approx (0.0990,\;0.7000,\;0.2010,\;1.4000)$, that significantly improves the uncontrolled case since it is characterized by the broadcasters at the desired level, a rather low level of inert individuals and a considerable level of self-information.

The temporal evolution of the controls *G*(*t*) in the forward and backward scenario is shown in Fig 4 (Bottom). In both scenarios, a stronger effort is required to convert an uncontrolled equilibrium superthreshold into a new Z-controlled equilibrium, with respect to the subthreshold one. This seems highly reasonable. In fact, while subthreshold cases would still lead to the survival of the campaign (even if under not so favorable conditions), the superthreshold equilibrium corresponds to a situation where the campaign has stopped, and therefore a considerably greater effort is needed to recover it. Fig 4 (Bottom) also reveals that the temporal evolution of the exerted controls is qualitatively different in the forward and backward scenarios suggesting that, between the two, the forward situation is definitely the easiest to manage. In this case, in fact, *G*(*t*) is a slowly increasing and saturating function that gradually drives the system trajectories toward the Z-controlled equilibrium. In the backward framework, the temporal trend of the control *G*(*t*) is instead much less predictable and reveals the complexity associated with this scenario, in which bistability or hysteresis-type behaviors – with a sudden collapse of the campaign – can occur. In this case, control first rapidly decreases until it reaches a minimum and then slowly increases to settle on a positive constant level.

### 4.2 Direct Z-control on the inerts

We now apply the Z-control to the uncontrolled model with the aim of decreasing the inerts to a desired value $i_{d}<i_{0}$. The parameters and the initial conditions are chosen as in Sect 4.1, namely: μ=0.05, ρ=0.25, λ=0.02, *p* = 0.7, *q* = 0.8, *a* = 0.5, *k* = 2, γ=0.2, *u*_0_ = 0.4, *b*_0_ = 0.5, *i*_0_ = 0.1, *m*_0_ = 1. For a better comparison with the previous subsection, we also chose ζ=0.03.

In correspondence of these parameter values, because of (16) and (17), one has: $i_{c}\approx 0.9231$ and $i_{d1}\approx 0.3394$. Furthermore, we set *i*_*d*_ = 0.01. For this choice, conditions $0<i_{d}<i_{0}<i_{d1}<i_{c}$ are verified so that Proposition 4 holds. Consequently, the Z-controlled model admits the unstable equilibrium $E_{1}\approx (0.9892,0.0008,0.0100,0.0017)$ and the equilibrium $E_{2}\approx (0.0778,0.9122,0.0100,1.8245)$ that is stable for all values of the parameters. Hence, we refer to *E*_2_ as the Z-controlled equilibrium.

**Fig 5:**
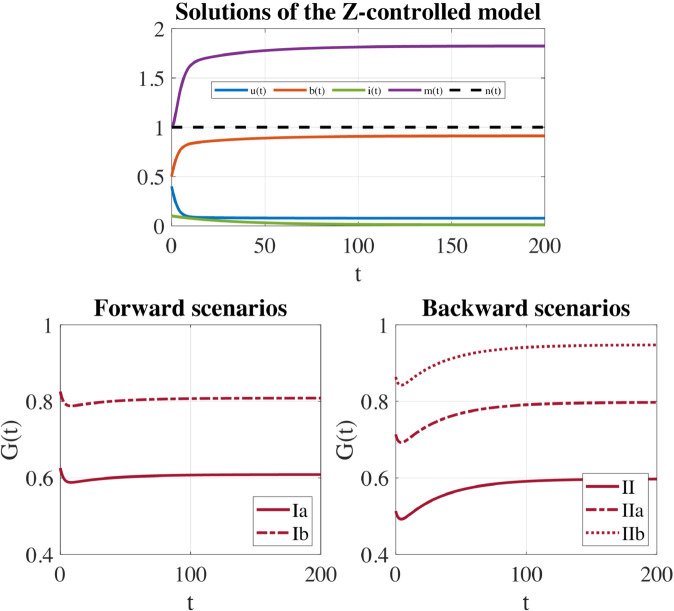
Z-controlled model: direct control on the inerts. Parameters: μ=0.05, ρ=0.25, λ=0.02, *p* = 0.7, *q* = 0.8, *a* = 0.5, *k* = 2, γ=0.2, ζ=0.03, and *i*_*d*_ = 0.01. Initial conditions: *u*_0_ = 0.4, *b*_0_ = 0.5, *i*_0_ = 0.1, *m*_0_ = 1. (Top) System trajectories approach the Z-controlled equilibrium $E_{2}\approx (0.0778,0.9122,0.0100,1.8245)$. (Bottom) Control functions *G*(*t*) applied to convert an uncontrolled attractor within the forward or the backward scenario into the new Z-controlled equilibrium. Left: Forward scenario - The subthreshold case (Ia) and the superthreshold case (Ib). Right: Backward scenario - The subthreshold case (II), the bistability case (IIa) and the superthreshold case (IIb).

Fig 5 (Top) shows that, for both forward and backward scenarios, the Z-control forces the dynamics of the system to approach the Z-controlled campaign-standing equilibrium *E*_2_, that considerably improves the uncontrolled case. *E*_2_ has in fact the inerts at the desired level, a very high level of the broadcasters and a considerable high level of self-information.

The temporal evolution of the controls *G*(*t*) is shown in Fig 5 (Bottom). In agreement with what we found for the Z-control of the broadcasters, also in this case, a stronger effort is required to convert an uncontrolled equilibrium superthreshold into the Z-controlled equilibrium for both the forward and backward scenario.

Unlike the Z-control of the broadcasters, when inerts are Z-controlled, the temporal evolution of the exerted controls is qualitatively similar in the forward and backward scenarios. In both of them, the control first decreases to a minimum and then increases to settle on a positive constant level. However, in the forward case, the long-term value of the control is slightly lower than the initial control value. In contrast, in the backward scenario, the long-term value of the control is higher than the value of the control initially exerted.

Lastly, a comparison between Fig 4 and 5 suggests that, when the inerts are Z-controlled, the control trends have less pronounced features but the overall magnitude of the applied control is greater compared to the case of the broadcasters’ control.

**Fig 6:**
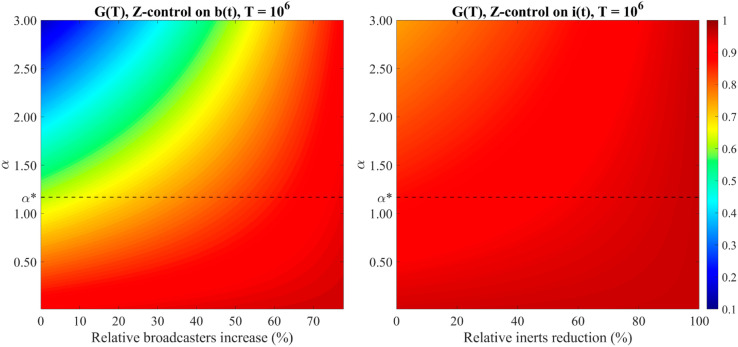
Z-controlled model. (a) Direct control on broadcasters. The color bar represents the stabilized value of *G*(*t*) after achieving a specific broadcasters increase for different values of *α*. Since *b*_*d*_ must satisfy $b_{d}<1\;-\;\frac{\mu }{\rho \;+\;\gamma k}\approx 0.8889$, the maximum broadcasters increase is about 77%. (b) Direct control on inerts. The color bar represents the stabilized value of *G*(*t*) after achieving a specific inerts reduction for different values of *α*. Parameters: μ=0.05, ρ=0.25, λ=0.02, *p* = 0.7, *q* = 0.8, *a* = 0.5, *k* = 2, γ=0.2, σ=0.95, ζ=0.03. Initial conditions: *u*_0_ = 0.4, *b*_0_ = 0.5, *i*_0_ = 0.1, *m*_0_ = 1. (For interpretation of the references to color in this figure legend, the reader is referred to the web version of this article).

This latter aspect is further emphasized in Fig 6 that summarizes the key features of a successful Z-controller when used to recover the campaign with increasing broadcasters or reducing inerts by a certain percentage. Within both the forward ($\alpha <\alpha^{*}$) and the backward ($\alpha >\alpha^{*}$) scenarios, we specifically focus on the supercritical case, namely when the referral campaign has stopped. We therefore consider α∈(0,3) and set σ=0.95. The other parameters are fixed as in Fig 4 and in Fig 5.

We recall that, for the chosen set of parameter values, the critical threshold $\alpha^{*}$ that separates the forward and backward scenarios is such that $\alpha^{*}=1.1684$ and is represented as a dashed black line in Fig 6. In particular, Fig 6 represents a contour plot of the final value of the Z-controller *G*(*t*) required by the Z-method to obtain (a) relative broadcasters increasing or (b) relative inerts reduction in the supercritical case for different values of *α*. We can see a marked distinction in the degree of control that must be applied, based on whether the objective is to encourage broadcasters or to reduce inertia.

When the control is aimed at increasing broadcasters, Fig 6(a) shows that for fixed values of $\alpha <\alpha^{*}$ (when the reactivated individuals are relatively few), the final value of *G*(*t*) varies very slightly with the percentage of broadcasters to increase and the degree of control required to recover the campaign is very high. On the contrary, for fixed values of $\alpha >\alpha^{*}$ (when the reactivated individuals are relatively high), the final value of *G*(*t*) increases progressively with the percentage of broadcasters to increase.

Moreover, when the percentage of broadcasters to increase is fixed, the final value of the control variable *G*(*t*) decreases for increasing values of *α* when the percentage is less than 50%, while it sets at a maximum effort level in the case of percentages above 50%.

Fig 6(b) instead demonstrates that reducing inertia, even by a small percentage, requires significantly more intensive control regardless of whether the amount of reactivated individuals is below (forward scenario) or above (backward scenario) the $\alpha^{*}$ threshold.

## 5 Exploratory analysis of bounded stochastic fluctuations

To complement the analysis of the uncontrolled and Z-controlled models, we now investigate the robustness of the system when the reactivation parameter *α* is subject to bounded stochastic fluctuations. This step is motivated by the fact that, in real socio-economic environments, parameters such as *α*, which governs the transition from indecision to informed adoption, are naturally affected by variations due to heterogeneity in information exposure, public sentiment or psychological factors.

The introduction of bounded stochastic perturbations in key model parameters has been shown to induce nontrivial dynamical phenomena, such as noise-induced transitions and phase-switching effects, in a variety of biological and physical systems [33]. A systematic analysis of different families of bounded stochastic processes, including the sine-Wiener process and the Tsallis Stariolo Borland process, is provided in [34,35], where their mathematical properties and relevance for modeling constrained but fluctuating dynamics are thoroughly discussed. For the purposes of the present study, we focus on the sine-Wiener process, which offers analytical tractability and a well-defined bounded support. The investigation of alternative formulations, such as the Tsallis Stariolo Borland dynamics, is left to future research.

The Sine-Wiener noise is constructed by applying the bounded sine function to a rescaled Wiener process *W*(*t*), resulting in


$$\chi (t)=B\sin\left(\sqrt{\frac{2}{\tau }}\;W(t)\right),$$

where B∈(0,1) controls the amplitude and τ>0 sets the correlation time. This process remains confined within (–*B*,*B*) and captures smoothly varying fluctuations with finite memory. The parameter *τ* controls the rate at which these fluctuations evolve: small values of *τ* correspond to rapidly varying (less persistent) noise, while larger values produce smoother, more persistent deviations. In this sense, *τ* represents the characteristic memory time of the socio-economic environment under consideration.

The fluctuation on the reactivation parameter *α* is then modeled as


$$\alpha (t)=\bar{\alpha }\left(1+\chi (t)\right),$$

where $\bar{\alpha }$ is the baseline level. As a result, α(t) varies within $\left(\bar{\alpha }(1\;-\;B),\;\bar{\alpha }(1\;+\;B)\right)$, remaining strictly positive and within a meaningful range.

This modeling choice reflects the idea that fluctuations in campaign reactivation effectiveness, for instance due to heterogeneous engagement, emotional saturation, or informational overload, are not uniform across different campaign regimes. Rather, they scale with the intensity of ongoing marketing activity.

Before analyzing the performance of the Z-control strategies under uncertainty, we investigate how the uncontrolled referral model (1) responds to bounded stochastic fluctuations in the reactivation rate α(t). The goal of this analysis is to explore the structural resilience of the campaign dynamics in the absence of active interventions. In particular, we aim to verify whether the system can autonomously withstand moderate variability in its key parameters, or whether external stabilizing actions are needed.

**Fig 7:**
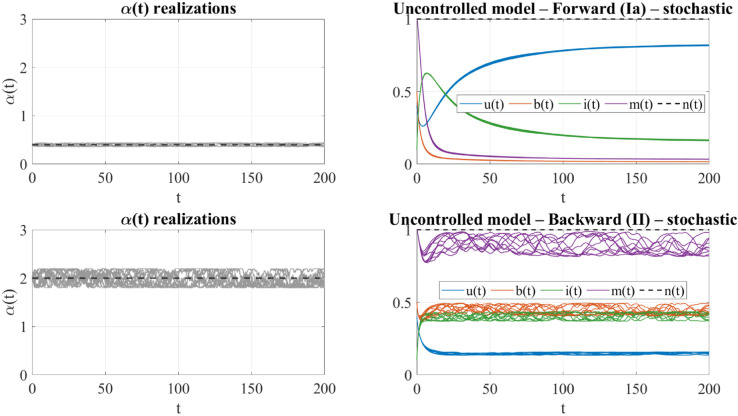
Uncontrolled model under stochastic fluctuations. Simulated trajectories for the forward (top) and backward (bottom) scenarios with stochastic reactivation rate α(t), modeled via a sine-Wiener process (*B* = 0.1, τ=10). Left panels show 10 realizations of α(t); right panels display the corresponding dynamics of *u*(*t*), *b*(*t*), *i*(*t*), *m*(*t*), and *n*(*t*). In the forward case ($\bar{\alpha }=0.4$), fluctuations are mild and dynamics remain stable. In the backward case ($\bar{\alpha }=2.0$), larger absolute variability leads to visible dispersion and increased volatility.

Fig 7 illustrates the behavior of the uncontrolled model under bounded stochastic fluctuations in the reactivation parameter α(t), generated via the sine-Wiener process with amplitude *B* = 0.1 and correlation time τ=10.

Fig 7 illustrates how bounded stochastic fluctuations in the reactivation rate α(t) affect the dynamics of the uncontrolled model in forward and backward regimes. Due to the multiplicative structure of the noise, the system’s response is strongly regime-dependent. In the forward scenario ($\bar{\alpha }=0.4<\alpha^{*}$), fluctuations in α(t) remain small in absolute terms and closely concentrated around the baseline value. As a result, the trajectories of *u*(*t*), *b*(*t*), *i*(*t*), and *m*(*t*) are tightly clustered and nearly indistinguishable from the deterministic case, indicating strong robustness to moderate uncertainty.

In contrast, in the backward scenario ($\bar{\alpha }=2.0>\alpha^{*}$), the same relative fluctuation level leads to much larger absolute variations in α(t). This results in visibly greater dispersion in the system’s response, particularly in *b*(*t*) and *i*(*t*), with some trajectories showing signs of collapse. These effects are due to the higher leverage of α(t) in this regime and the system’s proximity to bifurcation boundaries.

These findings support the interpretation that forward dynamics are inherently more robust to bounded uncertainty, while backward dynamics may require additional control mechanisms to mitigate stochastic instability.

Unlike the uncontrolled model, both Z-controlled systems do not explicitly depend on α(t), and therefore the dynamics remain unchanged across noise realizations. As a result, any variability in α(t) does not propagate to the state trajectories. Nevertheless, the control functions *G*(*t*) can exhibit some sensitivity to fluctuations.

**Fig 8:**
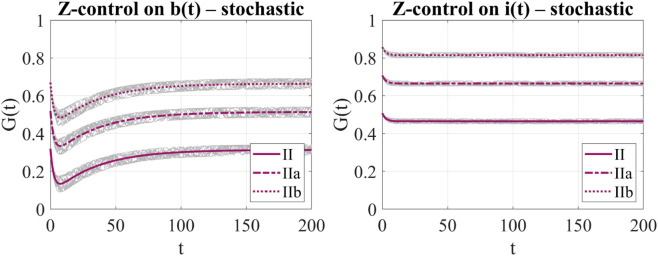
Control function G(t) under bounded stochastic fluctuations of α(t) (sine-Wiener noise with B=0.1, τ=10) in the backward regime ($\bar{\alpha }=2.0$), for the Z-controlled models. Left: control on *b*(*t*) (system (6)); Right: control on *i*(*t*) (system (14)). Scenarios are described in Sect 4.

Fig 8 illustrates the control function *G*(*t*) for the two Z-control strategies under bounded stochastic fluctuations of the reactivation rate α(t), modeled as a sine-Wiener process with amplitude *B* = 0.1 and correlation time τ=10. The simulation is performed in the backward regime ($\bar{\alpha }=2.0$), considering scenarios II, IIa, and IIb as defined in Sect 4. Each panel displays 10 sample paths of *G*(*t*) generated from independent realizations of the noise process, along with the corresponding deterministic reference.

In the left panel, which refers to Z-control applied to the broadcaster population *b*(*t*) (system (6)), we observe a wider spread in the stochastic realizations of *G*(*t*) defined in (4). The source of this variability lies in the term αpi(t), which appears explicitly in the control formula. By contrast, the right panel, which refers to Z-control applied to the inert population *i*(*t*) (system (14)), shows only mild variability in *G*(*t*) across noise realizations. Although the control function *G*(*t*), defined in Eq (12), also depends on the product αpi(t), in this case the state *i*(*t*) decays exponentially toward the target *i*_*d*_ = 0.1. As a result, the contribution of this term rapidly diminishes over time and remains significantly smaller than in the case of Z-control applied to *b*(*t*).

These results highlight that the sensitivity of the control function *G*(*t*) to stochastic fluctuations in α(t) is mediated by the dynamics of the informed population *i*(*t*). A persistently high *i*(*t*) amplifies the variability in *G*(*t*), while a rapidly decreasing *i*(*t*) dampens it, leading to more stable control signals.

## 6 Insights from the results

One of the most important aspects influencing the success of a referral marketing campaign is the consumer’s motivation, a factor that can be increased by incentives such as discounts, prizes, or exclusive benefits. In this respect, a widely quoted example is Dropbox’s referral program, which allowed the company to grow exponentially in just 15 months by offering free storage to users who invited friends to sign up. In this way, Dropbox turned its customers into active promoters of the service, ensuring that the brand spreads. Similarly, Airbnb encouraged users to share the service by using a referral system that offered a discount to both the sender and the receiver of the report, thus rapidly growing the customer base [36]. On the other hand, a common problem in referral campaigns is the loss of interest over time. This phenomenon, known as information overload, occurs due to an excess of advertising stimuli. To counteract it, many companies employ remarketing strategies as the delivery of advertisements to specific classes of customers, although these tools can generate negative reactions because of customers’ tendency to disregard repetitive corporate communications.

It is therefore evident that, in order for a referral marketing campaign to endure, companies have to face a really challenging task since appropriate marketing strategies must be not only employed, but they must also be carefully calibrated and strategically deployed over time.

When no control is applied to the referral model, reciprocal transition between the broadcaster and the inert class is a key mechanism for the survival of the campaign. And in particular, the reactivation rate from inerts to broadcasters - represented by the parameter *α* - is what needs to be most closely monitored. In fact, if only a few inert individuals are *reactivated* into broadcasters, the uncontrolled campaign will develop within a dynamical forward scenario. Otherwise, a dynamical backward scenario emerges, introducing the risk of hysteresis-type phenomena that - if not properly handled - can be responsible for sudden campaign collapses.

To increase the involvement of broadcasters while decreasing inertia, we introduced a time-dependent control function *G*(*t*) whose dynamics is not prescribed a priori. The application of the Z-control approach thus suggests which trend *G*(*t*) must have over time for the broadcasters or the inerts to be brought to a desired fixed level. To give a practical interpretation of the *G*(*t*) control, we stress that it must be able to act on the psychological and social factors that determine users’ behavior. The control *G*(*t*) can thus be understood as a kind of consistent communication that - removing psychological barriers and educating about the benefits of the program (through transparency, appropriate incentives and a good user experience) - is aimed at making the program simpler, more accessible and appealing, hence engaging a broader audience. We have applied the Z-control approach to control the referral dynamics for both the forward and the backward scenarios. We found that differences in the trend of the control over time emerge when (i) the objective of the control is to increase broadcasters or (ii) decrease inerts to a predetermined level.

In case (i), the forward scenario is certainly the most straightforward. In fact, when the reactivated inerts are not too much and the company wants to push the broadcasters to the desired level, marketing efforts *G*(*t*) must gradually increase with respect to an initial input until they settle at a constant value (slightly higher than the initial one). Z-controlling the broadcasters in a backward scenario, when a much larger quantity of reactivated inerts must be managed, instead requires marketing strategies *G*(*t*) with a less predictable trend over time than in the forward case. In fact, a non-monotic control *G*(*t*) must be now applied: after an initial input, a certain period of time follows in which marketing communications must quickly decrease to a minimum. This seems to be necessary so that the re-engaged individuals do not immediately feel overwhelmed. Subsequently, once psychological barriers have been mitigated and a sense of loyalty to the campaign has been cultivated, the application of marketing strategies can be progressively re-intensified until it stabilizes at a constant level (slightly lower than the initial one).

Interestingly in case (ii), when inerts are driven to a fixed value, marketing communications *G*(*t*) have to be non-motonic functions of time regardless of the quantity of inert individuals who are reactivated, that is for both forward and backward scenarios. In addition, the overall magnitude of the required control is much higher compared to case (i). This seems to validate what has already been widely observed in literature [37,38], namely that reducing inertia - albeit only marginally - is a considerable arduous task. In order to address this issue, companies will need to plan a more intensive use of marketing strategies, but carefully dosed over time. The non-monotonous form of these interventions *G*(*t*) would indeed seem essential to avoid excessive initial pressure and remove those psychological barriers usually associated with inertia. Immediate over-pressure could, in fact, cause a growing sense of irritation and a lack of interest that could progressively involve other customers as well. This, in turn, could lead to the failure of the campaign.

To account for uncertainty in consumer responsiveness, we introduced bounded stochastic fluctuations in the reactivation rate. This highlighted key differences between the two dynamical regimes from an economic perspective. Forward scenarios offer a low-risk environment: moderate marketing efforts lead to predictable outcomes, and campaign stability is preserved even under mild fluctuations. In contrast, backward scenarios are more fragile and investment-sensitive, with a risk of collapse that persists despite intensified efforts. Z-control helps stabilize both regimes, but its cost-effort profile depends on the target of intervention. Controlling broadcasters enables quicker improvements in visibility at relatively low initial cost, yet with greater variability in the effort required over time. Conversely, reducing inertia entails more consistent but higher investment, acting directly on behavioral barriers. This reveals a trade-off between agility and cost stability: while stimulating broadcasters boosts short-term diffusion, targeting inerts builds long-term engagement at a higher price.

A key distinction also emerges between the two types of intervention. Controlling broadcasters leads to faster visibility gains and lower initial costs, but with greater variability over time, especially in volatile settings. Controlling inerts, by contrast, ensures more stable trajectories and long-term engagement, but requires consistently higher effort. The trade-off is clear: stimulating broadcasters favors short-term reach with flexible strategies, while reducing inertia targets structural change at higher, more predictable cost.

According to these results, referral marketing initiatives might be strategically important in the shift to a circular economy, especially when they are led by reliable communicators like Greenfluencers. Although the model is theoretical in nature, it formalizes behavioral mechanisms that align with real-world dynamics of resistance, fatigue and re-engagement. This demonstrates how mathematical modeling can provide insightful qualitative information about the design of circular campaigns, helping in determining when and how to step in to preserve long-term consumer involvement in CE practices, build confidence and prevent overload.

## 7 Management implications and concluding remarks

Although this research presents an abstract mathematical framework, its application is specifically motivated by the behavioral challenges observed in CE transitions. CE items, such as remanufactured or reused goods, differ from traditional ones since they often need users to overcome skepticism or unfamiliarity and alter long-standing behaviors. Our model accounts for this psychological and social resistance through the class of inert individuals. Moreover, the suggested time-dependent interventions provide insight on how trust in CE models may be progressively increased and maintained over time. The non-monotonic feature of the controls, in particular, highlights how crucial it is to pace incentives and communication to prevent rejection or disengagement in the early stages. This makes the modeling approach pertinent to strategic planning in CE initiatives. Therefore, starting from the awareness that referral marketing can potentially serve as a highly effective tool in promoting circularity, in this paper we addressed questions as: Are there any warning signs that predict unexpected collapses in a referral campaign? What marketing strategies should be used to improve campaign’s performance? And how should these strategies be spread out over time to avoid boomerang effects? We have shown that, by combining bifurcation analysis and the Z-control approach, it is possible to obtain answers that can also offer operational management advices to companies and managers alike. In particular:

– The transition process from the inert to the broadcaster status requires rigorous monitoring. In fact, an excess of reactivated individuals in the campaign can cause hysteresis-type phenomena, which can lead to sudden campaign collapses if not managed properly. This aspect may be related to the fact that, in reactivated inert individuals, behavioral barriers may not have been fully eliminated. Such barriers may relate to both psychological factors (i.e. internal cognitive and emotional elements) and the social environment, with the latter emphasizing the significant impact of social structure and relationships on shaping consumer behavior [37]. Companies should therefore consider taking actions to specifically identify and reduce adoption barriers that consumers face daily, with the aim of encouraging greater support for the circular economy.
– To successfully promote circularity, it is crucial to assess how communication tools can effectively reduce perceptions of the consumer’s barriers [39]. And it is precisely in this perspective that the time-dependent Z-controller *G*(*t*) can be framed. Considered as a means to improve the performance of the campaign and to avoid possible sudden collapses, *G*(*t*) should be interpreted as a marketing strategy that company must appropriately apply over time with the aim of increasing the involvement of broadcasters and reducing inertia. Inspired by recent research [40–42], we suggest that the Z-controller *G*(*t*) could be identified with the Greenfluencers’ communication. Such specialized social media influencers use their platforms to raise environmental awareness and encourage sustainable living. Greenfluencers enjoy a high level of credibility, in part due to the perception that their actions are driven by deep intrinsic motivations rather than financial gain. As a result, their communications can effectively shape consumers’ perceptions of barriers to adopting pro-circularity practices, thereby reducing consumers’ inertia and encouraging pro-environmental behaviors [43]. In order for CE products, practices, or adoptions to become stably rooted in the population, companies should therefore plan to consider a strategic collaboration with Greenfluencers in order to educate consumers about initiatives of the companies in terms of circular economy.
– Greenfluencers’ communication and the related contents should, however, be appropriately balanced and distributed over time. The Z-control approach can in fact provide companies with qualitative indications that suggest how such marketing strategies must be applied over time in order to achieve the desired target. Specifically, the removal of inertia is the aspect of the process that presents the greatest challenge. A particularly intense communication effort should thus be directed by companies towards this, suitably distributed over time in a non-monotonous manner. This will ensure that consumers do not initially perceive the message as over-pressing, thereby avoiding the triggering of a boomerang effect.

This paper proposes an innovative application of the Z-control method as a means to enhance circular economy transition through a suitable green-oriented control of the referral marketing campaigns. The potential of referral marketing to boost confidence in the circular economy paradigm is in fact clear, given its capacity to influence consumer awareness, loyalty and participation in sustainable practices. We stress, however, that the methodological contribution of this work is theoretical, aiming to highlight dynamic mechanisms that influence the success or failure of referral marketing campaigns. Rather than providing context-specific recommendations, we focus on understanding how the timing and intensity of interventions can affect long-term outcomes.

Our findings also highlight the need for further exploration of the potential impact of emerging communication channels on the development of novel behavioral frameworks, such as those associated with the circular economy. In this sense, Greenfluencers are a notable and promising example. By encouraging a shift in mindset, they can foster the adoption of more sustainable choices by individuals, thereby disseminating these ideas to others and catalyzing a comprehensive transition of society towards a circular economy.

We consider this paper’s key concept to warrant additional investigation. There are various research lines that could be pursued to provide a more comprehensive exploration of the topic. (i) Evaluating the complementary use of optimization techniques as additional tools to promote circularity in the management of referral campaigns [44,45]. (ii) Considering the more involved framework of network-based models [46]: multilayer or multiplex networks have, in fact, the potential to reveal hidden patterns of influence, thus enhancing the qualitative suggestions provided through the presented approach. (iii) Investigating the emergence of on–off intermittency phenomenology in the model when some key control parameters undergo fluctuations driven by either stochastic or deterministic mechanisms [47,48]. (iv) Including spatial effects by extending the referral model within the framework of partial differential equations to study self-organization properties [49,50] or evaluate the impact of localized interventions [51,52].

We conclude by recalling that the interrogative form of the title wants to stress the exploratory nature of this work. Rather than providing a fully resolved claim, we aim to shed light on potential mechanisms by which well-managed referral campaigns could promote trust in circular economy efforts. We hope that this theoretical point of view will provide a conceptual framework for next empirical studies and practical interventions aimed at sustainable behavioral changes. At the same time, we also hope that this study might contribute to stimulating interest in the applications of mathematical modeling within the context of the circular economy, inspiring new lines of research in which the goals of circularity intersect with the tools of mathematics and the ever more pressing challenges of sustainability.

## Appendix A – Non-technical summary for interdisciplinary readers

This appendix aims to provide a conceptual non-technical overview of the main ideas and results of this paper, intended for readers from marketing, behavioral sciences and other interdisciplinary areas.

**The challenge.** Circular Economy (CE) initiatives often face behavioral barriers: consumers may be skeptical or reluctant to change habits. Referral marketing can help, as it makes use of the trust among peers to spread awareness. But in order for such campaigns to be successful, companies must carefully manage *how and when* to communicate. In fact, too much pressure can cause rejection, while too little may lead to collapse.

**The model in brief.** We model a population divided into three groups: *Unawares*, not yet informed about the campaign; *Broadcasters*, actively promoting the campaign; *Inerts*, informed but resistant or passive. People can be informed through referrals and gathering *self-information*, for example by means of personal research or social media. The model describes how individuals move among these groups over time.

**Z-Control.** Z-control is a dynamic approach designed to adjust marketing interventions based on the current state of the campaign. Instead of applying constant effort, it shows when to *intensify or reduce* marketing interventions (i.e., communication) to keep users engaged and avoid burnout.

**Main insights**

(i) *Non-linear strategies work best*: marketing initiatives should change over time rather than remain constant. After the first push, pull back and then re-engage.
(ii) *It is more difficult to target inertia*: converting inactive users is more difficult (and expensive) than assisting active ones.
(iii) *Early overload is risky*: it’s dangerous to communicate too much right away since it can backfire. Building trust takes time.
(iv) *Greenfluencers can help*: trusted voices with environmental credibility can maintain participation and reduce resistance. They can be seen as a practical example of the model’s control function.

**Why it matters.** These findings align with known patterns in behavioral science like decision fatigue, resistance to change and the importance of trust and social proof. Even though the model is theoretical, it can offer qualitative advice on how to structure communication in CE referral programs to promote trust and encourage long-term change. This conceptual framework could stimulate empirical studies by marketing practitioners, behavioral researchers and sustainability strategists alike.
